# Comments on “New Concerns for Neurocognitive Function during Deep Space Exposures to Chronic, Low Dose Rate, Neutron Radiation”

**DOI:** 10.1523/ENEURO.0329-19.2019

**Published:** 2020-01-02

**Authors:** Joseph J. Bevelacqua, James Welsh, S. M. Javad Mortazavi

**Affiliations:** 1 Bevelacqua Resources, Richland, Washington 99352; 2 Department of Radiation Oncology, Edward Hines Jr VA Hospital, Hines, Illinois 60141; 3 Medical Physics Department, Shiraz University of Medical Sciences, Shiraz, IR; 4 Diagnostic Imaging Department, Fox Chase Cancer Center, Philadelphia, Pennsylvania 19111

**Keywords:** HZE, LET, neurocognitive function, neutrons, radiation, space

## Abstract

Evaluations of the biological effects of space radiation must carefully consider the biological system response and the specific nature of the source term. Acharya et al. (2019) review neurocognitive function during deep space exposures to chronic, low dose rate, neutron radiation, but do not use a source term that reflects the actual space environment in terms of radiation types and their respective energies. In addition, important biological effects, including the adaptive response to the space radiation environment, are not addressed.

## Significance Statement

Acharya et al. (2019) review neurocognitive function during deep space exposures to chronic, low dose rate, neutron radiation, but do not use a source term that reflects the actual space environment in terms of radiation types and their respective energies. In addition, important biological effects including adaptive response to the space radiation environment are not addressed.

## 

This commentary addresses the article “New concerns for neurocognitive function during deep space exposures to chronic, low dose rate, neutron radiation” by Acharya et al. (2019). Considering the limitations of currently available technology for simulating the space radiation environment, this article (Acharya et al., 2019) outlines the use of a new neutron irradiation facility to simulate the low dose rates found in deep space. Their study showed neurobehavioral and electrophysiological defects in rodents subjected to continuous (6 month duration) exposure to low dose rate (1 mGy/d) neutron exposures. Despite the numerous strengths of this study, it has a few major shortcomings. The first shortcoming is due to ignoring the key point that in a realistic space environment, cells will be exposed to multiple low LET (linear energy transfer) protons before being traversed by intermediate and high-LET HZE (high charge and energy) particles. It is worth noting that a National Aeronautics and Space Administration report (Huff et al., 2016) clearly states that this sequential exposure can lead to the induction of adaptive responses in space that may significantly decrease the level of damages induced by high-LET HZE particles: “There have been several studies performed that indicate an adaptive response to low-dose ionizing radiation can provide a level of protection against future exposures (Bhattacharjee and Ito 2001; Mortazavi et al., 2003; Elmore et al., 2008, 2011; Rithidech et al., 2012). This may be particularly important for understanding risks in the space environment because the GCR [galactic cosmic radiation] environment is composed predominantly of protons, and it is realistic to expect that cells will be exposed to multiple hits of protons before being traversed by an HZE particle” (Huff et al., 2016). Moreover, an article authored by a large group of scientists from the United States, Canada, the United Kingdom, Russia, and Belgium have recently highlighted the cardinal role of adaptive response as an efficient method of biological protection in radiation risk reduction strategies for astronauts participating in space journeys (Cortese et al., 2018). It is worth noting that despite current controversies, some studies show cells pre-exposed to a low dose reveal decreased vulnerability to subsequent exposure to higher doses and produce a neuroprotective effect (Betlazar et al., 2016).

The second shortcoming is assuming that ^252^Cf neutron radiation can represent the biological effects of the HZE particles in a deep space mission. In ∼3% of decays, spontaneous fission occurs. This yields energetic fission products along with ∼3.75 neutrons per fission. The emitted neutrons are “fast” with a most probable energy of 0.7–1.0 MeV and an average energy of 2.1–2.3 MeV. Given this consideration, there are a number of issues with simulating solar particle events (SPEs) and GCR with a fission source, as follows:

1. The dominant dose component of ^252^Cf neutrons is not equivalent to more energetic protons and HZE particles. The energy differences are orders of magnitude apart (Bevelacqua, 2008, 2017).

2. The interactions from the neutrons include elastic and inelastic scattering and a limited number of reaction channels such as (n,gamma), (n,p), and (n,d; Bevelacqua, 1999, 2008, 2009, 2016). GCR and SPE open numerous higher-energy channels with the productions of pions, muons, and a host of spallation products and their associated hadronic cascades (Bevelacqua, 1999, 2008, 2009, 2016). The biological effects of these various species are not readily equated to a low-energy fission source.

3. In addition to direct reactions in tissue, the reactions with the spacecraft shell and components will vary significantly (Bevelacqua, 2008).

4. Characterizing a fission source in terms of delivered biological dose is significantly easier than determining the dose from a spectrum of protons and HZE particles of much greater energy (Bevelacqua, 2008).

5. The absorbed dose does not correspond to a biological detriment. This dose must be weighted with an appropriate factor [e.g., RBE (relative biological effectiveness), quality factor, or radiation weighting factor] to obtain a biological dose. Determining these factors is a nontrivial exercise (Bevelacqua, 2010). Using a stochastic International Commission on Radiologic Protection methodology (Bevelacqua, 1999, 2009), it is clear that energy dependence and radiation type are significant factors. Neutron factors are not the same as those for protons and HZEs.

## Synthesis

Reviewing Editor: Mark Baxter, Mount Sinai School of Medicine Decisions are customarily a result of the Reviewing Editor and the peer reviewers coming together and discussing their recommendations until a consensus is reached. When revisions are invited, a fact-based synthesis statement explaining their decision and outlining what is needed to prepare a revision will be listed below. The following reviewer(s) agreed to reveal their identity: NONE.

Equating the exposure of neutrons with low-LET and high-LET heavy charged particles is not as simplistic as equating cumulated dose exposures. There are complex biological and nuclear processes that differ based on radiation species, energy, dose-rate, etc., that can not be ignored when (presuming) a biological impact. The author elucidates these by providing clarification to the non-expert (e.g. nuclear physicist, etc.) on how and why the radiation species and energy is important to ascertaining the biological impact at the cellular scale.

The letter is well written and makes excellent points that may not be readily apparent to the non expert. The author justifies the shortcomings of the manuscript under question with sound scientific arguments. One change that should be considered is the language in lines 41-42 which details the decay mechanisms of 252Cf, explicitly stating that the majority by product are 6.1 MeV alphas. This information is not necessary to the argument presented, without further clarification, and could confuse readers when reading lines 48-49 (reason 1). Here the reader may not understand that these range out in less than 0.5 cm of air and may convolute the “dominant dose component of 252Cf neutrons...” with neutrons and alphas.
